# Intelligent Modelling of the Real Dynamic Viscosity of Rubber Blends Using Parallel Computing

**DOI:** 10.3390/polym15173636

**Published:** 2023-09-02

**Authors:** Ivan Kopal, Ivan Labaj, Juliána Vršková, Marta Harničárová, Jan Valíček, Hakan Tozan

**Affiliations:** 1 Department of Numerical Methods and Computational Modeling, Faculty of Industrial Technologies in Púchov, Alexander Dubček University of Trenčín, Ivana Krasku 491/30, 020 01 Púchov, Slovakia; ivan.kopal@tnuni.sk (I.K.); ivan.labaj@tnuni.sk (I.L.); juliana.vrskova@tnuni.sk (J.V.); 2 Department of Electrical Engineering, Automation and Informatics, Faculty of Engineering, Slovak University of Agriculture in Nitra, Tr. A. Hlinku 2, 949 76 Nitra, Slovakia; jan.valicek@uniag.sk; 3 Department of Mechanical Engineering, Faculty of Technology, Institute of Technology and Business in České Budějovice, Okružní 10, 370 01 České Budějovice, Czech Republic; 4 College of Engineering and Technology, American University of the Middle East, Egaila 54200, Kuwait; hakan.tozan@aum.edu.kw

**Keywords:** rubber blends, curing process, intelligent modelling, generalised regression neural network, parallel computing

## Abstract

Modelling the flow properties of rubber blends makes it possible to predict their rheological behaviour during the processing and production of rubber-based products. As the nonlinear nature of such complex processes complicates the creation of exact analytical models, it is appropriate to use artificial intelligence tools in this modelling. The present study was implemented to develop a highly efficient artificial neural network model, optimised using a novel training algorithm with fast parallel computing to predict the results of rheological tests of rubber blends performed under different conditions. A series of 120 real dynamic viscosity–time curves, acquired by a rubber process analyser for styrene–butadiene rubber blends with varying carbon black contents vulcanised at different temperatures, were analysed using a Generalised Regression Neural Network. The model was optimised by limiting the fitting error of the training dataset to a pre-specified value of less than 1%. All repeated calculations were made via parallel computing with multiple computer cores, which significantly reduces the total computation time. An excellent agreement between the predicted and measured generalisation data was found, with an error of less than 4.7%, confirming the high generalisation performance of the newly developed model.

## 1. Introduction

The vulcanisation, cross-linking, or curing of rubber blends (RBs) is one of the most crucial technological processes in the rubber industry. Throughout this process, the rheological behaviour of the vulcanised material undergoes changes attributed to the formation of a spatial molecular network among the individual polymer chains [1]. Rheological measurements conducted during cross-linking reactions enable the detection of these changes, and the obtained results find application in various practical tasks, such as the design, modelling, simulation, optimisation, and automation of the production process, assessment of material processability and stability, quality control, and more [2]. The formation of the cross-linked spatial structure during the curing process modifies the rigidity of the blend. Consequently, monitoring the variations in modulus over the curing time delivers continuous insights into the curing process and offers an understanding of the method and degree of cross-linking. These aspects have fundamental significance within the rubber industry [3].

The Rubber Process Analysers (RPAs) are currently the most powerful devices available for measuring and analysing changes in the rheological behaviour of RBs during the curing process. These RPAs assess the stiffness changes of the RB test sample over time by subjecting them to sinusoidal shear-loading at a specific angular frequency and cure temperature [4]. The results obtained from RPA measurements are typically interpreted in the form of isothermal rheological cure curves (vulcanisation or rheometric curves) or as a functional relationship between the elastic (storage) torque (proportional to the shear modulus of elasticity or modulus of rigidity) and the curing time at a constant temperature [5]. Analysing the cure curves allows for the direct determination of essential curing characteristics such as scorch time, optimum curing time, minimum torque, maximum torque, and torque difference. Furthermore, it facilitates the calculation of various derived characteristics, including curing speed ratio, cure rate index, degree of cross-linking, thermo-plasticity, and reversion time [6]. Due to their capacity to provide a wide range of technologically significant information, cure curves have become the most commonly employed method for interpreting RPA measurements in routine rubber practice, as well as in the targeted modification of existing materials, and in the research and development of new polymer-based materials [7].

The results obtained from RPA measurements can also be interpreted by establishing a functional relationship between actual dynamic viscosity (RDV) and curing time. RDV represents the real component of the complex dynamic viscosity and serves as an indicator of the material’s resistance to flow or deformation under external shear forces [8]. To calculate RDV, the ratio of the loss (viscous) shear modulus to the applied oscillation frequency is determined during the curing process at a given cure temperature [5,8]. RDV–time curves offer a more detailed analysis of the curing process compared to dynamic complex viscosity–time curves and cure curves [3]. They exhibit a significantly higher sensitivity, particularly towards changes in the rheological behaviour of RBs resulting from variations in the chemical cross-linking bonds during curing [4]. When combined with cure curves, RDV–time curves provide a more comprehensive understanding of the curing progress, which is important, especially when studying the extrusion and injection moulding of elastomeric mixtures [9]. As a result, they have become a focal point of interest in the present study.

Curing process modelling plays an essential role in both conventional rubber production and the development of new rubber-based materials. This enables the efficient optimisation of RBs’ processability, a prediction of their rheological behaviour under different curing conditions, and estimation of the final performance of vulcanisates. However, due to the highly sophisticated, complex, and nonlinear nature of RB curing, precise mathematical treatments using traditional analytical methods are exceedingly challenging, if not impossible [10]. Although several ad hoc models have been reviewed and are available, for instance, in works [10,11,12], they consider different aspects of curing independently or describe individual phases in a self-consistent manner. Currently, a complete analytical model that encompasses all the features of RB curing does not exist in practice. The use of artificial neural networks (ANNs) can prove highly beneficial in modelling complex processes like these, as they have the unique ability to establish any nonlinear relationships among numerous variables without prior knowledge of the processes or system models [13]. In fact, several studies have explored the application of ANNs in the modelling and forecasting of curing processes for RBs. For example, in the work [14], an advanced ANN model was developed to predict vulcanisation data for various commercially available RBs used in tire production. Study [15] employed an ANN approach to analyse the dependency of rheometric properties on RB components. In article [16], three distinct ANN architectures were introduced to forecast the optimal curing time for different RBs at varying cure temperatures. The publication [17] compares various machine learning methods to predict the rheometric properties of RBs. In article [18], the ANN was used to provide predictions of vulcanisation characteristics based on a comprehensive database of RBs with diverse compositions. In the aforementioned works, as well as in a number of other studies, predictive models of RB curing have been formulated based on ANN analysis of a set of experimental cure curves, which serve as representative network patterns. However, to the best of our knowledge, an ANN modelling of RB curing based on RDV–time curves has not been extensively explored in the literature to date. Therefore, in the presented study, a novel ANN model was proposed to predict RDV–time curves of RBs with varying compositions cured under different temperature conditions. The flexible architecture of the model, developed using the Generalised Regression Neural Network, allows for researchers to extend its application to other aspects (input and output variables), not only for RBs but also for polymers in general.

### Generalised Regression Neural Network Theory

Various types and architectures of ANNs are widely recognised as one of the most powerful tools in artificial intelligence for modelling complex nonlinear phenomena [13]. As a result, they find extensive application in various practical fields [19]. Currently, the most commonly used type of ANN is the multi-layer, feed-forward ANN, trained using the error back-propagation learning algorithm (BPNN) [20,21]. However, BPNN has limitations. It requires a high number of iterations to converge to the desired solution, tends to fall into local minima during network optimisation, and is sensitive to initial weights and biases, as well as the setting and iterative tuning of several training parameters during the training process [13,19,20,21]. In order to overcome these limitations, a new ANN concept called the Generalised Regression Neural Network (GRNN) was introduced by Specht in 1991 in [22]. Since this type of ANN is the focus of the presented work, we provide a brief description in the following lines of this section.

The GRNN is a memory-based, supervised, probabilistic type of feed-forward ANN. It utilises a radial basis activation function in the hidden layer and has a simple, highly parallel, dynamic structure. It possesses a strong ability to nonlinearly map any continuous functions between input and output vector variables [7]. The network’s straightforward structure and computational implementation have led to its extensive use in various fields, particularly when solving function approximation or regression problems [23]. The most significant advantage of GRNN over BPNNs is its considerably faster one-pass training algorithm. This algorithm eliminates the need for iterative procedures during supervised learning, allowing for the function estimate to be drawn directly from the training data, without prior knowledge of the specific functional form [24]. Instead, the unknown function is represented as a conditional probability density function between independent and dependent variables, which can be empirically determined from the observed dataset using a Parzen–Rosenblatt density estimator with a specified Gaussian kernel bandwidth [25]. Consequently, the probabilistic prediction of the dependent variable for a given independent variable is unique and not reliant on the training procedure or initial conditions, as is the case with BPNNs [26].

**Figure 1:**
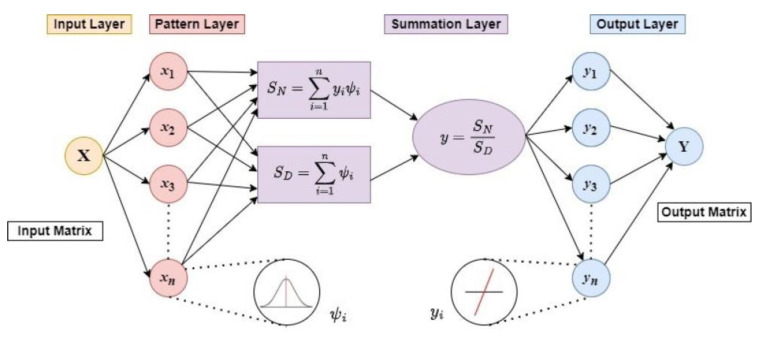
Schematic diagram of GRNN general topological structure.

The concept of the GRNN is based on the theory of nonlinear regression analysis [27], and its structure consists of four neuron layers, input, pattern (radial basis), summation, and output (linear) layer, as shown schematically in Figure 1 [13]. (The software implementation of the GRNN general topological structure can be found in our previous work [7].)

The GRNN is a feed-forward ANN, so the signals always propagate from the first neuron layer to the last one [13]. The number of neurons in the input and output layers corresponds to the number of independent (input) and dependent (target) variables *x*, *y* of the network, respectively. The input layer is fully connected to the pattern layer, where the number of hidden neurons is equal to the number of *n* input training samples *x_i_*. The Gaussian Radial Basis Activation Function (RBF) or Gaussian RBF kernel [25] using (1)

(1) $$\psi_{i}\left(x,x_{i}\right)=\sum_{i=1}^{n}exp\left(-\frac{D_{i}^{2}\left(x,x_{i}\right)}{2\sigma^{2}}\right),$$

in each pattern layer neuron is centred on each training sample *x_i_*, which is then stored in the neuron memory. Its output is a measure of the squared Euclidean distance *D_i_* of the current input vector element *x* from each training input vector sample *x_i_*, that is computed using the Formula (2) [27].

(2) $$D_{i}^{2}\left(x,x_{i}\right)=\sum_{i=1}^{m}{\left\|x-x_{i}\right\|}^{2}=\sum_{i=1}^{m}{\left(x-x_{i}\right)}^{T}\left(x-x_{i}\right).$$

Each pattern layer neuron is connected to the two neurons in the summation layer: the *S_N_*—summation neuron, which computes the sum of the weighted outputs of the pattern layer (3)

(3) $$S_{N}=\sum_{i=1}^{n}y_{i}\psi_{i},$$

and the *S_D_*—summation neuron, which is used to compute the unweighted sum of the output of each pattern layer neuron (4)

(4) $$S_{D}=\sum_{i=1}^{n}\psi_{i}$$

where the weights *y_i_* are the target training vector values.

The output layer divides the output of the *S_N_*—summation neuron by that of the *S_D_*-summation neuron to obtain the predicted value $\hat{y}$ of the target vector *y* as (5):

(5) $$\hat{y}=\frac{S_{N}}{S_{D}}.$$

After substituting Equations (1)–(4) into Equation (5), the regression of a dependent variable *y* on an independent variable *x*, which is an estimate of the most probable value $\hat{y}\left(x,\sigma \right)$ at a given *σ*, can be presented in the form of Nadaraya–Watson kernel estimator of the regression function as (6) [28]:

(6) $$\hat{y}\left(x,\sigma \right)=\frac{\sum_{i=1}^{n}y_{i}exp\left(-\frac{\sum_{i=1}^{m}{\left(x-x_{i}\right)}^{T}\left(x-x_{i}\right)}{2\sigma^{2}}\right)}{\sum_{i=1}^{n}exp\left(-\frac{\sum_{i=1}^{m}{\left(x-x_{i}\right)}^{T}\left(x-x_{i}\right)}{2\sigma^{2}}\right)}.$$

Equation (6) shows that the regression of a dependent variable *y* on an independent variable *x*, or conditional mean value of $\hat{y}\left(x,\sigma \right),\;$is a nonlinearly weighted average of all training target values *y_i_* for training input cases *x_i_*, with the weighting depending on the Euclidean distance of training sample *x_i_* from the point of prediction *x* and Gaussian RBF kernel shape *σ*, usually called the smoothing factor or spread constant. When a new pattern is presented to the network, that input pattern is compared to all of the stored patterns in the training set to determine how far it is from those patterns. The output that is predicted by the network is a proportional amount of all of the outputs in the stored training set, whereas the proportion is based on how far the new pattern is from the given patterns in the training set. It is also clear from Equation (6) that the spread constant *σ* is the only unknown parameter in the network affecting the fitness of the GRNN architecture that needs optimisation.

The spread constant *σ* determines if the conditional mean $\hat{y}\left(x,\sigma \right)$, or GRNN model given by Equation (6), works as an approximator (large *σ* values will straighten the path of the model line) or as an interpolator (small *σ* values essentially create a dot-to-dot map). However, if the value of *σ* is too big, it degrades the fitting error (network underfitting phenomena). In contrast, a too-small value of *σ* can degrade the GRNN’s ability to generalise and may generate ineffective predictions (network overfitting phenomena) [28]. At the same time, it represents the standard deviation of *x* from *x_i_* in the Gaussian RBF kernel, so the GRNN can produce optimal results with variance *σ*^2^ as long as the *σ* value is no greater than this standard deviation. Therefore, the optimised *σ* is theoretically not unambiguous, and must be found empirically in the process of the network training so that the error between the target and predicted data is minimal [13].

Generally, GRNN training represents the optimisation of its spread constant using various optimisation methods [29,30]. A trial-and-error method was, for example, used to determine the spread constant *σ* of the GRNN model to predict the curing characteristics of RBs in our earlier work [7]. A cross-validation method for the GRNN optimisation was employed in article [31], which was dedicated to the identification of material parameters in the constitutive model of hyper-elastic materials such as rubber. A hold-out method of selecting the optimal value of *σ* was proposed in [22]. In [32], a plug-in algorithm and a cross-validation procedure based on traditional mathematical methods, including the theory of kernel density estimators, as well as a nature-inspired optimisation approach known as the particle swarm optimisation method, is described. Article [33] presents an extensive review of the research conducted on the optimisation of ANNs, including GRNNs, through genetic algorithms of artificial intelligence searches. In [22], the GRNN-training algorithms employing data dimensionality reduction techniques, such as K-means clustering and principal component analysis (increasing the model performance), are discussed. The presented study describes a novel training algorithm that optimises the GRNN *σ* value for modelling RDV–time curves of RBs with varying CB filler contents vulcanised at different cure temperatures. The algorithm utilises a novel optimisation procedure and parallel computing technique, which leverages multiple computer cores to perform multiple operations simultaneously [34,35]. This approach dramatically reduces the total time required for the network training compared to the commonly used algorithms mentioned above.

## 2. Materials and Methods

### 2.1. Materials

The composition of the investigated RBs, the function of individual ingredients in them, and their manufacturers are provided in Table 1.

### 2.2. Samples Preparation

The blends were prepared using a two-stage mixing in accordance with ISO2393 [36] in a laboratory mixer, Brabender Plastograph EC plus (Brabender GmBG & Co.KG, Duisburg, Germany), with an electrically heated chamber volume of 80 cm^3^. In the initial three minutes, the rubber matrix of the blend was masticated at a temperature of 90 ± 1 °C and a rotor speed of 50 ± 1 rpm. Then, the ZnO was added and mixed for 45 s, followed by the CB, which was mixed for 3 min, and finally the stearic acid, which was mixed for 30 s. The mixed blend was further homogenised at a temperature of 70 ± 1 °C using a laboratory two-roll mill, LaboWalz W150 (Voght Labormaschinen GmBH, Berlin, Germany), with a cylinder diameter of 150 mm, length of 400 mm, a working gap between the rolls of 1.5 mm, a roll speed of 24 rpm, and a gear ratio of 1:1.4. After homogenisation, the blends were allowed to rest for 24 h at laboratory temperature. In the second mixing stage, the rested blends were mixed for 3 min under the same conditions as in the first mixing stage. After this duration, S and TBBS were added, with each ingredient mixed for 1.5 min. The mixed blends were again homogenised on the two-roll mill under the same conditions as in the first mixing stage, and then left to rest for 24 h at laboratory temperature before subsequent rheological analysis.

### 2.3. Rheological Analysis

The rubber process analyser RPA 2000 (Alfa Technologies Ltd., Akron, OH, USA) was used to collect the experimental time dependence RDV curves of the investigated RBs with varying CB filler contents: 0 phr, 15 phr, and 30–75 phr (with a constant increment of 5 phr). In order to assess the impact of curing temperature on the RDV values, each blend underwent isothermal rheological tests conducted at constant temperatures ranging from 165 °C to 210 °C (with a constant increment of 5 °C). The oscillating angle of the rheometer disk was set to 1°, and the oscillating frequency was set to 1.67 Hz.

### 2.4. Artificial Neural Network Modelling

The GRNN model was implemented in the MATLAB^®^ software package, Version R2016a 64-bit (win64), with the inclusion of the Neural Network Toolbox and Parallel Computing Toolbox (MathWorks, Natick, MA, USA). These toolboxes offer all the necessary resources for efficient work with ANNs and parallel computing. The MATLAB^®^ software package was installed on a personal computer running Windows 10; Intel^®^ Core^TM^ i5-12450H, CPU^@^ 2.4 GHz, 16 GB RAM, 64-bit; SSD 250 GB; GPU: NVIDIA GeForce GTX 1650, 6 GB.

## 3. Results and Discussion

### 3.1. Experimental Results

**Figure 2:**
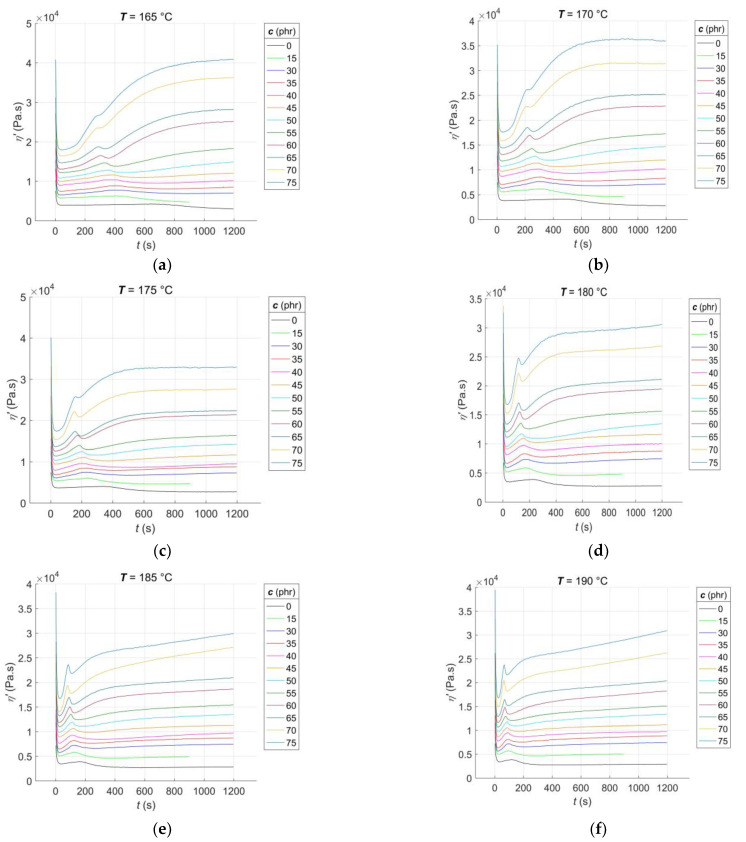

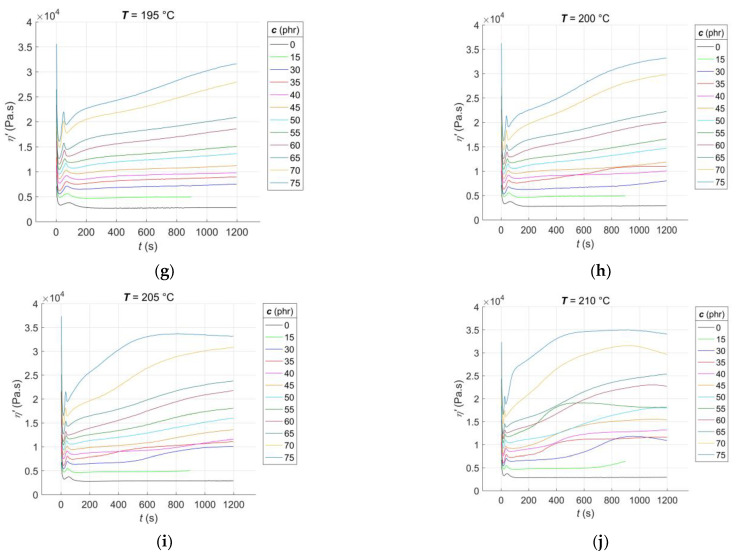
Experimental RDV–time curves of RBs with varying contents of CB filler, vulcanised at different temperatures: (**a**) 165 °C; (**b**) 170 °C; (**c**) 175 °C; (**d**) 180 °C; (**e**) 185 °C; (**f**) 190 °C; (**g**) 195 °C; (**h**) 200 °C; (**i**) 205 °C; (**j**) 210 °C.

The RDV–time curves *η*’(*c*, *T*, *t*) for the RBs with CB filler contents of *c* = [0, 15, 30:5:75] phr, vulcanised at constant cure temperatures of *T* = [165:5:210] °C, are presented in Figure 2.

From Figure 2a–j, it is evident that, much like the rheological cure curves, the RDV–time curves can be segmented into several parts that correspond to different phases of the curing process [1]. The initial segment of the curve occurs immediately after the sample is placed in the rheometer measuring chamber. During this stage, the blend is pre-heated, and the sample temperature gradually equilibrates with the temperature of the rheometer chamber. Additionally, the preliminary chemical reactions that occur during the induction stage of curing [37] are initiated. As time progresses during the curing process, the temperature of the sample rises, causing the blend’s resistance to flow during its cyclic shear loading to decrease, resulting in a decrease in the RDV value. The rate of this decrease is dependent on both the CB contents and the cure temperature. In accordance with the recipe, increasing the CB contents leads to a decrease in the rubber content of the blend. This, in turn, accelerates the pre-heating process of the blend to a constant temperature that is uniformly distributed throughout the sample volume while simultaneously decreasing the RDV value [38]. With an increase in the cure temperature, the rate of pre-heating in the blend accelerates while the RDV value decreases. Additionally, when both the CB contents and cure temperature increase, the local minimum of the RDV–time curve at this stage of the test tends to shift towards lower times. However, it is important to note that the homogeneity of the individual samples plays a crucial role in this trend. A lower degree of dispersion of the filler in the blend can result in non-uniform pre-heating, leading to the non-monotonic nature of this trend [39].

After reaching its local minimum, corresponding to the scorch time of the blend *t*_s1_ [2], the RDV–time curve begins to increase due to the formation of a spatial molecular network, leading to an increase in the blend’s resistance to flow. At this stage of the test, a peak appears on the RDV–time curve that is not visible on the cure curve [7]. The peak is the result of two competing and parallel processes: the formation and breakdown of poly-sulfidic cross-links, creating more sulphur radicals. These radicals subsequently allow for the formation of not only mono- and di-sulfidic cross-links but also un-cross-linked pendant sulfidic groups [40]. The peak position shifts almost monotonically towards lower times with an increase in CB content and cure temperature, as the activators and accelerators of vulcanisation enter the network formation process slightly earlier each time [41]. The slopes of the peak correspond to the rate of the networking process. Until the peak is reached, the formation of new poly-sulfidic cross-links dominates, which is the reason for the increase in the RDV value. However, after the peak is exceeded, their breakdown dominates, accompanied by a decrease in the RDV, which continues until a local minimum is reached at this stage of the test, with a tendency to shift towards lower times. As poly-sulfidic cross-links are thermally unstable, the rate of their breakdown increases with an increase in cure temperature [42]. At CB contents above 15 phr and cure temperatures of 170 °C or higher, the RDV–time curve starts to increase again after reaching its local minimum. This increase is due to the predominance of the formation process of more solid and thermally stable mono- and di-sulfidic cross-links over the breakdown of poly-sulfidic ones. The uncrosslinked pendant sulfidic groups also contribute to the increase in blend resistance to flow, naturally leading to an increase in RDV.

At cure temperatures above 200 °C in the final stage of the test, the CB contents in the blend and the degree of uniformity of its pre-heating during the induction stage of curing determine the type of cure that occurs in RBs. Depending on these factors, cure to no equilibrium (with an incomplete cross-linking process), cure to equilibrium (with a completed cross-linking process), and cure to a maximum RDV with reversion (with the continued process of breaking sulfidic cross-links) can be observed [43].

### 3.2. Data Pre-Processing for Neural Network Computations

In order to develop a predictive GRNN model of RDV–time curves for RBs with different contents of CB filler cured at various temperatures, the CB content *c*, cure temperature *T*, and curing time *t* were used as input data [7]. The corresponding values of RDV *η*’(*c*, *T*, *t*) served as the target data of the network. The raw experimental data registered before the onset of the rise of the individual RDV–time curves (Figure 2a–j) do not represent the networking process itself, but only the pre-heating of the blend and the temperature-initiated preliminary chemical reactions occurring during the induction stage of curing [4]; therefore, these data points were excluded from the ANN analysis.

The input and target data were arranged into the *Inputs* = [*c*; *T*; *t*] and *Targets* = [*η’*] data matrices, respectively, which can be presented in the shortened matrix form as:

(7) $$\mathrm{Inputs}={\left({inputs}_{ij}\right)}_{m,n}={\left(\begin{array}{c} c_{ij}^{k}\\ T_{ij}^{k,l}\\ t_{ij}^{k,l} \end{array}\right)}_{3,q}$$

and

(8) $$\mathrm{Targets}={\left({targets}_{ij}\right)}_{m,n}={\left(\eta_{ij}^{\prime k,l}\right)}_{1,q},$$

where *i* = 1, 2, ..., *m* and *j* = 1, 2, ..., *n* represent the row and column index of the input/target matrix, respectively; *k* = 1, 2, ..., length(*c*), *l* = 1, 2, ..., length(*T*) and *q* = 1, 2, ..., length(*t*) are the index of the element of the vector of CB contents *c*, cure temperatures *T* and curing times *t*, respectively, where length(*c*) and length(*T*) represent the lengths of the respective vectors, while

(9) $$length\left(t\right)=\sum_{p=1}^{kxl}length\left(t_{1,q_{p}}^{k,l}\right)$$

is the sum of the lengths of the vector *t* for CB contents *k* and cure temperature *l*, and *m* is the number of input/target variables.

The min–max normalisation technique was used to rescale all the input and target data to fall within the interval [0, 1] while preserving their original distribution. This ensures that the training of the network is not biased by variables with significantly larger value ranges while also partially reducing the influence of outliers in the noisy experimental data. The min–max normalisation was carried out according to the following formula [44]

(10) $$x_{norm}=\frac{x-x_{min}}{x_{max}-x_{min}},$$

where *x*_norm_ is the normalised data, while *x*, *x*_min_ and *x*_max_ are the original data, and their minimum and maximum values, respectively. The normalised data can be reversed to the original data after GRNN simulation according to the following relationship [44]

(11) $$x=x_{min}+x_{norm}\left(x_{max}-x_{min}\right).$$

**Figure 3:**
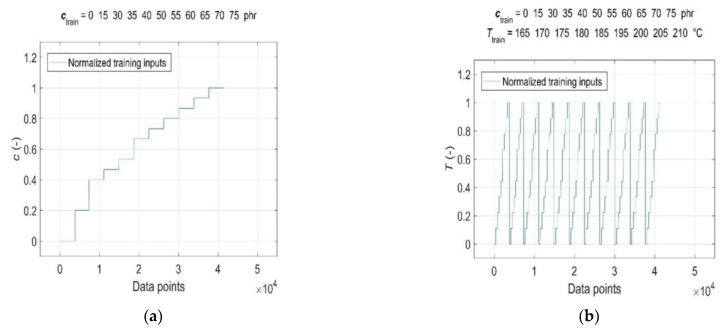

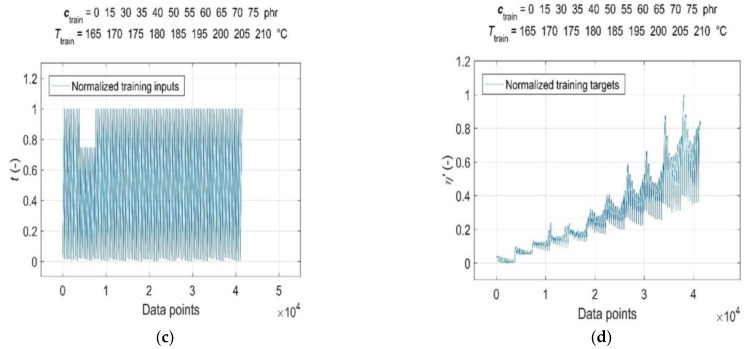
Normalised training inputs for: (**a**) CB contents *c*_train_ = [0, 15, 30:5:40 50:5:75] phr in RB; (**b**) cure temperatures *T*_train_ = [165:5:185, 195:5:210] °C; (**c**) curing time *t*_train_ and (**d**) normalised training targets *η*’(*c*_train_, *T*_train_, *t*_train_).

**Figure 4:**
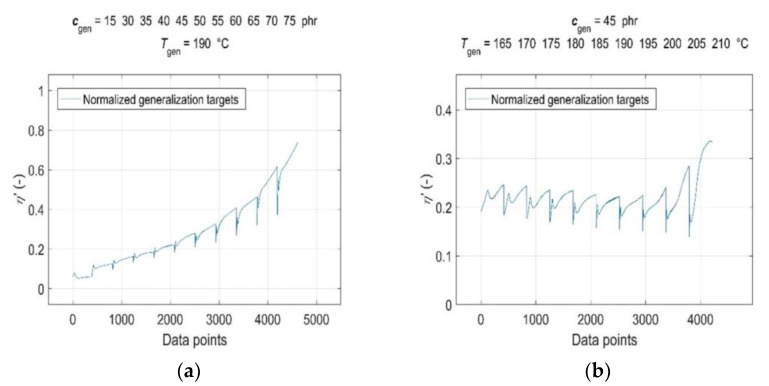
Normalised generalisation targets *η*’(*c*_gen_, *T*_gen_, *t*_gen_) for: (**a**) CB contents *c*_gen_ = [15, 30:5:75] phr in RB at cure temperature *T*_gen_ = 190 °C and (**b**) cure temperatures *T*_gen_ = [165:5:210] °C at CB contents *c*_gen_ = 45 phr in RB.

Consequently, both the normalised input and target data were split into a training dataset, representing a set of representative patterns used for training the GRNN, and two tests or generalisation datasets, which were used to evaluate the predictive performance and generalisation ability of the trained model [19]. The CB contents of *c*_train_ = [0, 15, 30:5:40 50:5:75] phr, cure temperatures of *T*_train_ = [165:5:185, 195:5:210] °C, corresponding curing time values of *t*_train_ and RDV values of *η’*(*c*_train_, *T*_train_, *t*_train_) were used for training the GRNN. The range of representative training data was selected to correspond to the ranges of CB contents in RB and cure temperatures commonly used in rubber processing, taking into account the technological, economic and environmental aspects of the production process [1,9]. The remaining data of *c*_gen_ = 45 phr at *T*_gen_ = [165:5:210] °C, *c*_gen_ = [15, 30:5:75] phr at *T*_gen_ = 190 °C, corresponding values of *t*_gen_ and *η*’(*c*_gen_, *T*_gen_, *t*_gen_), which were not included in the training dataset, were selected to evaluate the predictive performance and generalisation ability of the model. Since the sample without a CB filler (*c*_gen_ = 0 phr) served only as a reference frame when training the GRNN; it was not used in testing. In contrast, a sample with a CB content of 15 phr with a significantly shorter curing time registered at all temperatures was incorporated into the ANN analysis in order to investigate the effect of its length on the forecasting skills of the trained model. Normalised training inputs and targets, as well as normalised generalisation targets, are presented in Figure 3 and Figure 4, respectively.

### 3.3. GRNN Training Algorithm for Parallel Computing

As mentioned above, the essence of the GRNN training process lies in the optimisation of spread constant *σ* as the sole adjustable parameter that directly influences the network’s predictive success and determines its generalisation performance. Commonly used methods to find the optimal value of *σ* involve systematically searching the state space of possible solutions to minimise an appropriate cost function, typically by iteratively reducing the sum-squared error (*SSE*) or mean-squared error (*MSE*) between the predicted and target datasets at different values of *σ_i_*. Generally, during the GRNN-supervised training on representative training patterns, an iterative search is performed for the value of *σ,* at which the *SSE* (*MSE*) between the trained network’s predicted and measured generalisation data is minimised. However, this approach places significant demands on computational time, memory, and computer performance, especially when dealing with problems with a large number of high-dimensional, nonlinear training patterns (as in our case). Additionally, it requires a time- and memory-consuming iterative solution to the problem of network overfitting—where the developed GRNN model, while accurately fitting the training targets, may fail to reliably predict generalisation data—and the problem of network underfitting—where the model’s performance is poor on both training and generalisation data [13].

In order to improve the efficiency of the parameter *σ* optimisation process, we proposed and successfully implemented a new and powerful GRNN training algorithm in this study. This algorithm requires significantly fewer iterations, and with repeated computations performed within each iteration, harnesses the abilities of fast parallel computing, utilizing all available cores of a modern computer’s multi-core processor [34,35]. In contrast to the commonly used optimisation methods mentioned earlier, the proposed algorithm does not aim to minimise the error between the trained network’s predicted and target generalisation data. Instead, in the interval [*σ*_min_, *σ*_max_], it searches for *σ* from the population of its expected values *σ_i_*, where the maximum of the mean absolute percentage error (*MAPE*) between the modelled and real training data is smaller than a pre-specified value and, from a practical point of view, a sufficiently small value of *Err*_max_. Once the algorithm finds a suitable *σ* value, a trained GRNN is simulated using generalisation inputs, leading to a significant reduction in the total number of computations or computation time. The error value *Err*_max_, along with the range of the interval of expected *σ_i_* values and the step of their successive selection *s*, allows for efficient regulation of the network’s balance between overfitting and underfitting, without requiring multiple time-consuming retrainings. Furthermore, the implementation of parallel computing techniques in repetitive computations ensures the simultaneous and processor-controlled utilisation of all available computational resources (cores) of the utilised computer, resulting in a further substantial reduction in computational time.

The proposed GRNN parallel training algorithm automates the solution of the network training optimisation problem, which is formulated as the following model:

(12) $$\hat{y}\left(x,\sigma \right)=\hat{y}\left(x,arg\underset{\sigma_{i}}{\max}\left(MAPE\left(y,\hat{y},\sigma_{i}\right)<{Err}_{max}\right)\right)=\frac{S_{N}\left(x,y,\sigma \right)}{S_{D}\left(x,\sigma \right)},$$

for

(13) $$\sigma_{i}=\sigma_{min}:\;s\;:\sigma_{max},where\;i=1,2,\ldots ,m,$$

and

(14) $$MAPE=\frac{1}{n}\;\sum_{j=1}^{n}\left|\frac{y_{j}-{\hat{y}}_{j}}{y_{j}}\right|\cdot 100,$$

where *x*, *y*, $\hat{y}$ are training inputs, measured and predicted training targets with *n* elements, respectively; *σ*_min_ and *σ*_max_ are the bounds of the interval of assumed *m* values of the *σ_i_* of parameter *σ* chosen with step *s*, while the quantities *S_N_* and *S_D_* are the outputs of the GRNN summation neurons as described above. A MATLAB^®^ code of parallel computing implementation for GRNN training algorithm (12) is presented in Algorithm 1 below:
**Algorithm 1.** MATLAB^®^ code for GRNN parallel training algorithm% Setting the spread constant population vector with step *s* Spread = [Spread_min:s:Spread_max]; % Parallel computing loop for calling the built-in GRNN training function parfor ii = 1:length(Spread)   pop_GRNN(ii).net = newgrnn(Inputs_train,Targets_train,Spread(ii)); end % Calling the built-in simulation function of the trained GRNN with % training inputs in parallel computing mode for ii = 1:length(Spread)    Outputs_train = sim(pop_GRNN(ii).net,Inputs_train,...     ‘useParallel’,’yes’);
  % Calculation of the absolute error of the trained network  Err = Targets_train − Outputs_train;
  % Calculation of the average absolute percentage error of the trained   % network  pre_MAPE = abs(Err./Targets_train);  mean_MAPE = mean(pre_MAPE(isfinite(pre_MAPE))) * 100;
  % Conditional storage of the corresponding variables in the pop_GRNN  % structure   if mean_MAPE < max_MAPE    pop_GRNN(ii).Spread = Spread(ii);    pop_GRNN(ii).Outputs = Outputs_train;     pop_GRNN(ii).MAPE = mean_MAPE;   else
    % Premature termination of the cycle    break  end end % Identification of the MAPE maximum value index in the pop_GRNN % structure [~,k] = max([pop_GRNN.MAPE]);
% Setting the variables of the found values of the corresponding % parameters if ~isempty([pop_GRNN(k).Spread])  spread = pop_GRNN(k).Spread;  net_GRNN = pop_GRNN(k).net;  Outputs_GRNN_train = pop_GRNN(k).Outputs;
  % Removing empty fields from the pop_GRNN structure  pop_GRNN = pop_GRNN(1:length([pop_GRNN.Spread]),:); else
  % Terminate execution  return end

A necessary and sufficient condition for running Algorithm 1 in the parallel computing mode is that the computations in the individual operations in the parfor loop with integer steps are independent of the results of the computations in the previous iterations so that they can be distributed among all available processor cores and executed simultaneously. Otherwise, the parfor loop, as well as network simulations using the sim instruction, with the “useParallel” parameter disabled, will run as they do in traditional serial data processing with a single computational resource [34]. If the PC used, or its software, does not allow for parallel computing (older versions of MATLAB^®^ and versions without the Parallel Computing Toolbox installed), removing the prefix par from the parfor command, as well as the “useParallel” and ”yes/no” parameters from the sim command, will ensure the normal mode of serial computations. However, the GRNN training algorithm will then be much slower—the computational time required to train a network with a fitting error *Err*_max_ of less than 1%, and then simulate the optimised GRNN model on the training data with the parameter *σ* sought by Algorithm 1 within the interval of its expected values [0.014, 0.015] with a selection step of 0.001, amounts to approximately 170 s, while the time required to simulate the optimised model on the generalisation data amounts to approximately 5 s. Using the technique of parallel computation on the four available physical cores of the processor of the cooperating computer, described in Section 2.4, the time required to train the network and then simulate the optimised model on the training data was reduced to approximately 62 s almost three times, while the time required to simulate the optimised model on the generalisation data remained almost unchanged. It is natural to expect that a processor with a higher number of physical cores would result in further increases in computational speed. In testing, it has been shown that, compared to the standard training algorithm, which, in each training iteration, minimises the *SSE* between predicted and target generalisation data without the use of parallel computing techniques, the computation time of Algorithm 1 is more than 10 times shorter.

### 3.4. Goodness-of-Fit Model Evaluation

A quantitative evaluation of the goodness-of-fit of the GRNN model (12) to the training data was performed using the basic statistical accuracy metrics [45], namely using the linear regression coefficient or correlation coefficient

(15) $$R=\frac{cov\left(y,\hat{y}\right)}{s_{y}s_{\hat{y}}},$$

where $cov\left(y,\hat{y}\right)\;$ is the covariance and $s_{y},s_{\hat{y}}\;$ are the standard deviations of the variables $y,\hat{\;y}$, respectively:

mean absolute error:

(16) $$MAE=\frac{1}{n}\;\sum_{j=1}^{n}\left|y_{j}-{\hat{y}}_{j}\right|,$$

mean squared error:

(17) $$MSE=\frac{1}{n}\;\sum_{j=1}^{N}{\left(y_{j}-{\hat{y}}_{j}\right)}^{2},$$

root mean squared error:

(18) $$RMSE=\sqrt{\frac{1}{n}\;\sum_{j=1}^{n}{\left(y_{j}-{\hat{y}}_{j}\right)}^{2}}.$$

Mean absolute percentage error *MAPE* is defined by relation (14).

Mean of residuals is calculated by relation (19)

(19) $$MOR=\frac{1}{n}\;\sum_{j=1}^{n}\left(y_{j}-{\hat{y}}_{j}\right),$$

and residuals by (20)

(20) $$Training\;Errors=y_{j}-{\hat{y}}_{j}.$$

**Figure 5:**
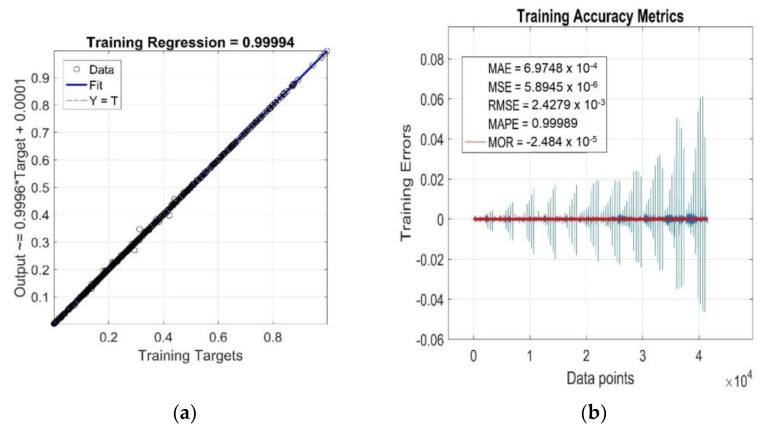
Statistical goodness-of-fit metrics of the GRNN model to the entire training dataset: (**a**) *R*; (**b**) *MAE*, *MSE*, *RMSE*, *MAPE*, *MOR* and *Training Errors*.

The results of the goodness-of-fit model evaluation to the training data are shown in Figure 5a,b. Since the GRNN model was optimised in the process of global network training off all the training data, all the observed accuracy metrics presented in these figures were calculated for the entire training dataset, not for the individual RDV–time curves *η*’(*c*_train_, *T*_train_, *t*_train_) of the investigated RB with different CB contents *c*_train_ at different cure temperatures *T*_train_.

**Figure 6:**
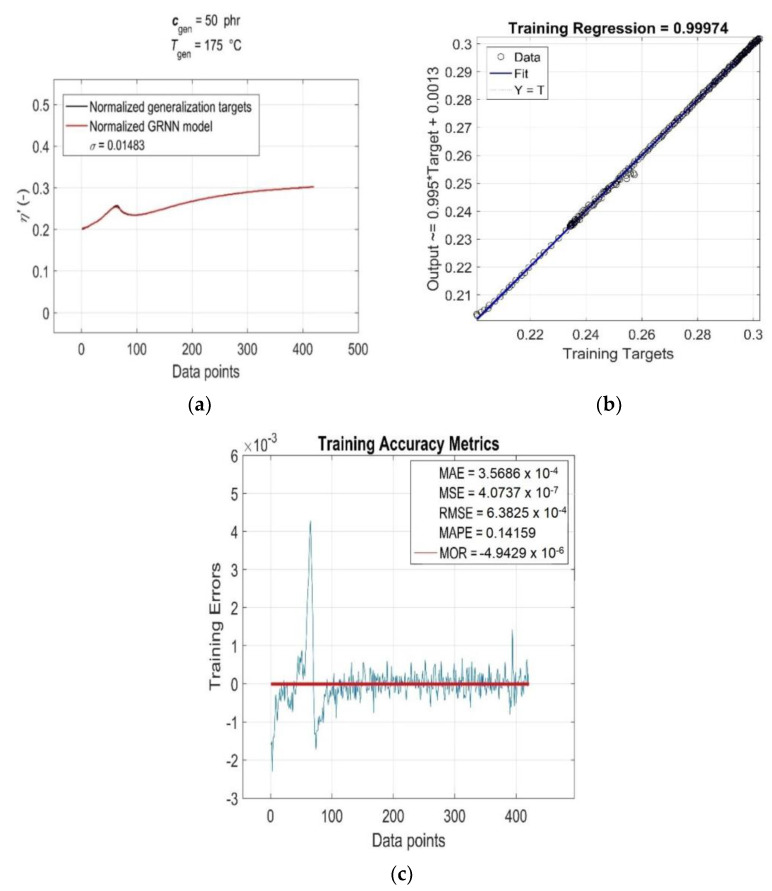
(**a**) GRNN simulation results and accuracy metrics of the trained model for the RDV–time curve with *c*_train_ = 50 phr at *T*_train_ = 175 °C: (**b**) *R* and (**c**) *MAE*, *MSE*, *RMSE*, *MAPE*, *MOR* and *Training Errors* (blue line).

As can be seen from Figure 5a, the value of the linear correlation coefficient *R =* 0.99994 is very close to +1, and the best linear regression (solid line) practically overlaps the perfect linear fit line (dotted line), which indicates a very strong positive correlation between the targets and outputs of the training data. The relatively very low parameter values *MAE* ≅ 6.97 × 10^−4^, *MSE* ≅ 5.89 × 10^−6^, *RMSE* ≅ 2.43 × 10^−3^ and *MAPE* ≅ 1 (Figure 5b) indicate a low mean of residuals, low variance and a low standard deviation, as well as a low mean of absolute percentage model error, respectively. The residuals of the individual RDV–time curves (*Training Errors*) are relatively uniformly distributed around the near-zero line, with outliers mainly concentrated in the lower part [45], which slightly reduces the degree of reliability of the optimised GRNN model. With the increasing content of CB filler in RB and increasing cure temperature, the variance values of residuals exhibit a strong non-monotonic trend (Figure 5b), but this is not indicative of their high heteroscedasticity [45]. In fact, for a single constant value of *c*_train_ and *T*_train_, the only independent variable in the analysed functional dependence *η*’(*c*_train_, *T*_train_, *t*_train_) is the curing time variable *t*_train_, which has very little effect on the distribution of residuals and their variance values (excluding a few outliers corresponding to local extrema), as demonstrated in Figure 6. This figure displays the simulation results of the trained network and the accuracy metrics of the trained GRNN model computed for the RDV–time curve with *c*_gen_ = 50 phr at *T*_gen_ = 175 °C, which was chosen randomly.

From the above analysis of training accuracy metrics, it can be concluded that the fitting performance of the GRNN model (12) on the training data is very satisfactory, with a mean of residuals *MOR* ≅ −2.48 × 10^−5^ (red line) and mean absolute percentage error *MAPE* less than the required 1% (Figure 5b).

### 3.5. Model Generalisation Capability and Forecasting Accuracy Evaluation

The evaluation of the generalisation capability and forecasting accuracy of the developed GRNN model was performed on two normalised generalisation datasets corresponding to all CB contents, except *c*_gen_ = 0 phr, at a cure temperature of 190 °C, and to all cure temperatures at *c*_gen_ = 45 phr of CB contents in RB, which were not included in the training data.

**Figure 7:**
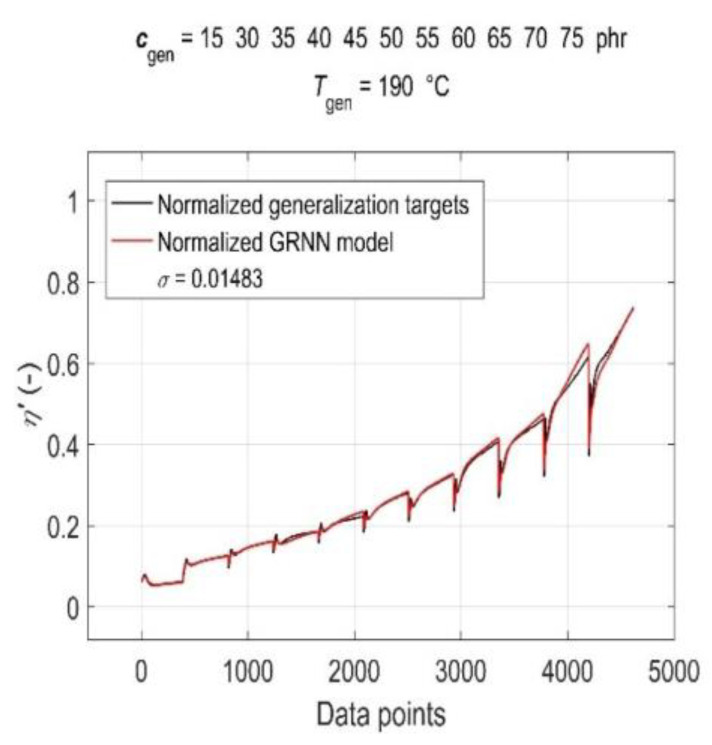
Simulation results of the trained GRNN for all CB contents in RB, except 0 phr, at a cure temperature of 190 °C in the form of a graphical comparison of normalised generalisation targets and normalised model outputs.

**Figure 8:**
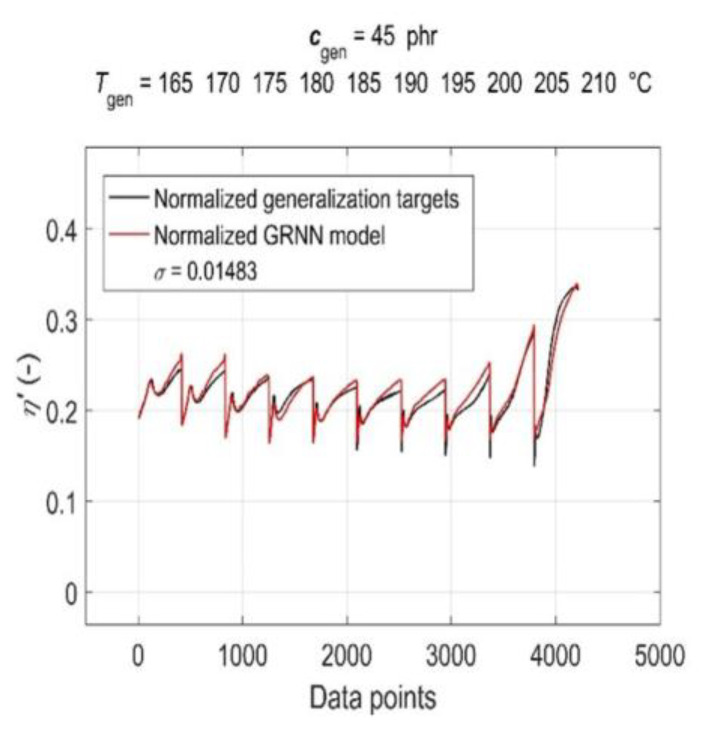
Simulation results of the trained GRNN for all cure temperatures at 45 phr of CB contents in RB in the form of a graphical comparison of normalised generalisation targets and normalised model outputs.

The simulation results of the trained GRNN with both generalisation datasets are presented in Figure 7 and Figure 8 as a graphical comparison of normalised generalisation targets and normalised model outputs. From the above figures, it can be seen that due to the global training of GRNN on the entire training dataset, the prediction confidence level for different values of generalisation *c*_gen_ and *T*_gen_ varies, with the GRNN trained model being slightly more sensitive to changes in *T*_gen_ at a constant *c*_gen_ than to changes in *c*_gen_ at a constant *T*_gen_.

**Figure 9:**
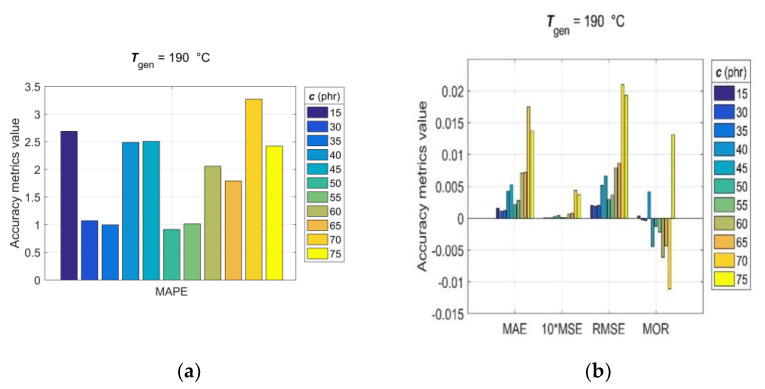
Accuracy metrics of the trained GRNN model for individual RDV–time curves with *c*_gen_ = [15, 30:5:75] phr at *T*_gen_ = 190 °C: (**a**) *R*, *MAPE* and (**b**) *MAE*, *MSE*, *RMSE*, *MOR*.

**Figure 10:**
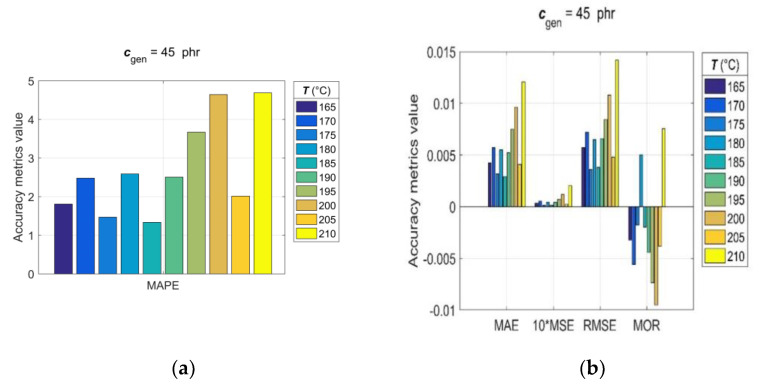
Accuracy metrics of the trained GRNN model for individual RDV–time curves with *c_gen_* = 45 phr at *T_gen_* = [165:5:210] °C: (**a**) *R*, *MAPE* and (**b**) *MAE*, *MSE*, *RMSE*, *MOR*.

Since, for rubber practice, it is essential to know the prediction accuracy for each single predicted RDV–time curve, the statistical accuracy metrics (14)–(20) were computed with generalisation data simulated individually for each pair of *T*_gen_/*c*_gen_ and *c*_gen_/*T*_gen_. The values of the observed accuracy metrics, divided into two groups based on their corresponding magnitudes, are presented in the bar charts shown in Figure 9 and Figure 10.

The analysis of accuracy metrics for individual predicted RDV–time curves showed that *R* values range from 0.9683 to 0.9959, *MAE* from 2.9 × 10^−3^ to 12.1 × 10^−3^, *MSE* from 1.22 × 10^−3^ to 20.3 × 10^−3^, *RMSE* from 3.5 × 10^−3^ to 14.3 × 10^−3^, *MOR* from −9.5 × 10^−3^ to 7.5 × 10^−3^, and *MAPE* from approximately 1 to 4.7, which confirms the very good predictive performance and generalisation ability of the developed GRNN model. At this point, it is appropriate to note that the discrepancy between fitting error (*Err*_max_) and prediction error (*MAPE*) is a direct consequence of the optimal balance between underfitting and overfitting achieved in the GRNN training process in the process of optimizing the GRNN model (6) by the training algorithm (12).

The raw experimental data, data prepared for visualisation, and MATLAB^®^ scripts enabling their detailed viewing are available at [46].

## 4. Conclusions

The presented work is dedicated to the intelligent modelling of the rheological properties of rubber blends during their vulcanisation.

A series of 120 real dynamic viscosity–time curves obtained by a Rubber Process Analyser for styrene–butadiene rubber blends with varying carbon black contents vulcanised at different temperatures was analysed.

A novel, highly efficient Generalised Regression Neural Network model was developed to predict the changes in the real dynamic viscosity of the investigated rubber blends during their vulcanisation process.

The optimisation of the model was performed through a novel training algorithm based on an iterative search for such a value of the Generalised Regression Neural Network spread constant that the fitting error of the target training data was less than a pre-set, from a practical point of view, sufficiently small value of 1%.

All repeated computations in the training process, as well as all network simulations, were performed through parallel computing using all available cores of a multi-core computer’s processor. This approach made it possible to significantly reduce the total computational time required for very satisfactory network training and model optimisation.

A clearly noted MATLAB^®^ code of the developed parallel computing training algorithm for Generalised Regression Neural Network is presented.

An excellent agreement was found between the predicted and measured generalisation data, with an error of less than 4.7%, confirming the high generalisation performance of the optimised model.

The presented study demonstrates the potential of intelligent modelling to predict the rheological properties of rubber blends. This approach can find practical applications in the design and optimisation of production processes within the conventional rubber industry. Additionally, it holds significant promise for research and development efforts focused on creating new materials and composites based on rubbers or elastomers in general. It is also possible to use the Generalized Regression Neural Network Model presented in this paper to conduct experimental studies on polymers, as it can be used to accurately predict the results of unrealized tests from the entire range of representative training data.

## Figures and Tables

**Table 1:** Composition of studied rubber blends.

Material	Contents (phr)	Producer	Function
Styrene–butadiene rubber grade 1500	100	Synthos Kralupy a.s., Kralupy nad Vltavou, Czech Republic	Matrix
Carbon black type N550 (CB)	0, 15, 30–75 *	Makrochem Sp. z o.o., Lublin, Poland	Filler
Zinc oxide (ZnO)	3	SlovZink a.s., Koseca, Slovakia	Vulcanisation activator
Stearic acid	1	Setuza a.s., Ústí nad Labem, Czech Republic	Vulcanisation activator
Sulfur Crystex OT33 (S)	1.75	Eastman Chemical Company, Kingsport, TN, USA	Vulcanising agent
TBBS **	1	Duslo a.s., Šaľa, Slovakia	Vulcanisation accelerator

* 30–75 with a steady increase of 5 phr (parts per hundred rubber) in CB filler ** N-tertiarybutyl-2-benzothiazole-sulfenamide.

## Data Availability

Datasets related to this article can be found at https://data.mendeley.com/datasets/h37ktpsdz4/1, an open-source online data repository hosted at Mendeley Data (Kopal, I., Labaj, I., Vršková, J., Harničárová, M, Valíček, J., Tozan, H., 2023).
